# Antimicrobial resistance in livestock: advances and alternatives to antibiotics

**DOI:** 10.1093/af/vfy001

**Published:** 2018-04-19

**Authors:** Ronald R Marquardt, Suzhen Li

**Affiliations:** 1 Department of Animal Sciences, The University of Manitoba, Winnipeg MB, Canada; 2 All Natural Nutritional Products (ANNP) Inc., The University of Manitoba Smartpark, Winnipeg MB, Canada

**Keywords:** antibodies, antibiotic resistance, bacteriophages, nanoparticles, vaccines

**Implications**

- Antibiotic microbial resistance, as reported by the American Medical Association, is considered to be one of the greatest threats to human health.
- Advances in biotechnology have demonstrated that the efficacy of antibiotics can be restored by the use of antibiotic-peptide conjugates.
- Recently, several highly effective and alternative means of treating and controlling disease caused by microorganisms have been developed. This includes the use of a new gene editing technology (CRISPR/Cas9), genetically modified bacteriophages, engineered peptides, nanoantibiotics, improved vaccines, highly effective chicken and plant immunoglobulins, and Eubiotics. Improved DNA technology has greatly facilitated the selection of livestock that have genetic resistance to pathogenic microorganisms.
- Scientific breakthroughs in disease control will be able to safely overcome the problem of antibiotic resistance. More research, development, and evaluation, worldwide, is required.

## Introduction

### Antibiotic Resistance: Possible Threats to Human Health

Antibiotic microbial resistance is considered to be one of the greatest threats to human health. In the United States, more than 2 million people are infected with antibiotic resistant bacteria annually, with 23,000 deaths as a direct result (Hampton, 2013). The O’Neil commission reviewed means to counteract the global threat of antibiotic resistance (O’Neil, 2016). It predicted that by 2050, 10 million deaths world-wide will be attributable to antimicrobial resistance. In addition to increased resistance to existing agents, there is a lack of new antibiotics in development. The commission made the following recommendations to reduce the consumption of antibiotics.

1. Implement a massive global public campaign to improve global awareness of antimicrobial resistance.
2. Improve hygiene and prevent the spread of infection.
3. Reduce unnecessary use of antimicrobials in agriculture and their dissemination into the environment.
4. Improve global surveillance of drug resistance and antimicrobial consumption in humans and animals.
5. Promotes new, rapid diagnostics to cut unnecessary use of antibiotics.
6. Promote development and use of vaccines and alternatives.

A committee of the European Medicines Agency and the European Food Safety Authority outlined measures that could be implemented to reduce the use of antimicrobial agents in animal husbandry in the European Union, and its resulting impact on food safety (EMA and EFSA, 2017). The recommended options (non-prioritized) included the following:

1. Develop national strategies for monitoring antimicrobial use and AMR development.
2. Establish national targets for antimicrobial use reduction.
3. Use of on-farm health plans.
4. Increase the responsibility of veterinarians for prescribing antimicrobials.
5. Increase the availability of rapid and reliable diagnostics.
6. Improve husbandry and management procedures for disease prevention and control.
7. Rethink livestock production systems to reduce inherent disease risk. Possible recommended alternatives to antibiotic use include probiotics and prebiotics, competitive exclusion, bacteriophages, immunomodulators, organic acids, and teat sealants.

### The One Health Commission

One Health is a “collaborative effort of multiple disciplines -working locally, nationally, and globally – to obtain optional health for people, animals and our environment.” Antimicrobial resistance is one of the most important issues that epitomizes the principles of One Health. Integrated approaches to reduce selection pressure and disrupt antimicrobial resistance transmission cycles on a global scale must be sought that are founded not only on sound One Health principles, but also based on economic evidence and on principles of social equity and global access to effective healthcare for people and their animals. An international agreement would help ensure the global coordination needed to accomplish these aims (Robinson et al., 2016).

The goal of the current review is to provide an overview of recent advances and alternatives to the use of antibiotics by the animals feed industry.

### Use of the CRISPR/Cas Gene Editing System to Reverse Antibiotic Resistance and as an Antimicrobial Agent

**Figure 1.**
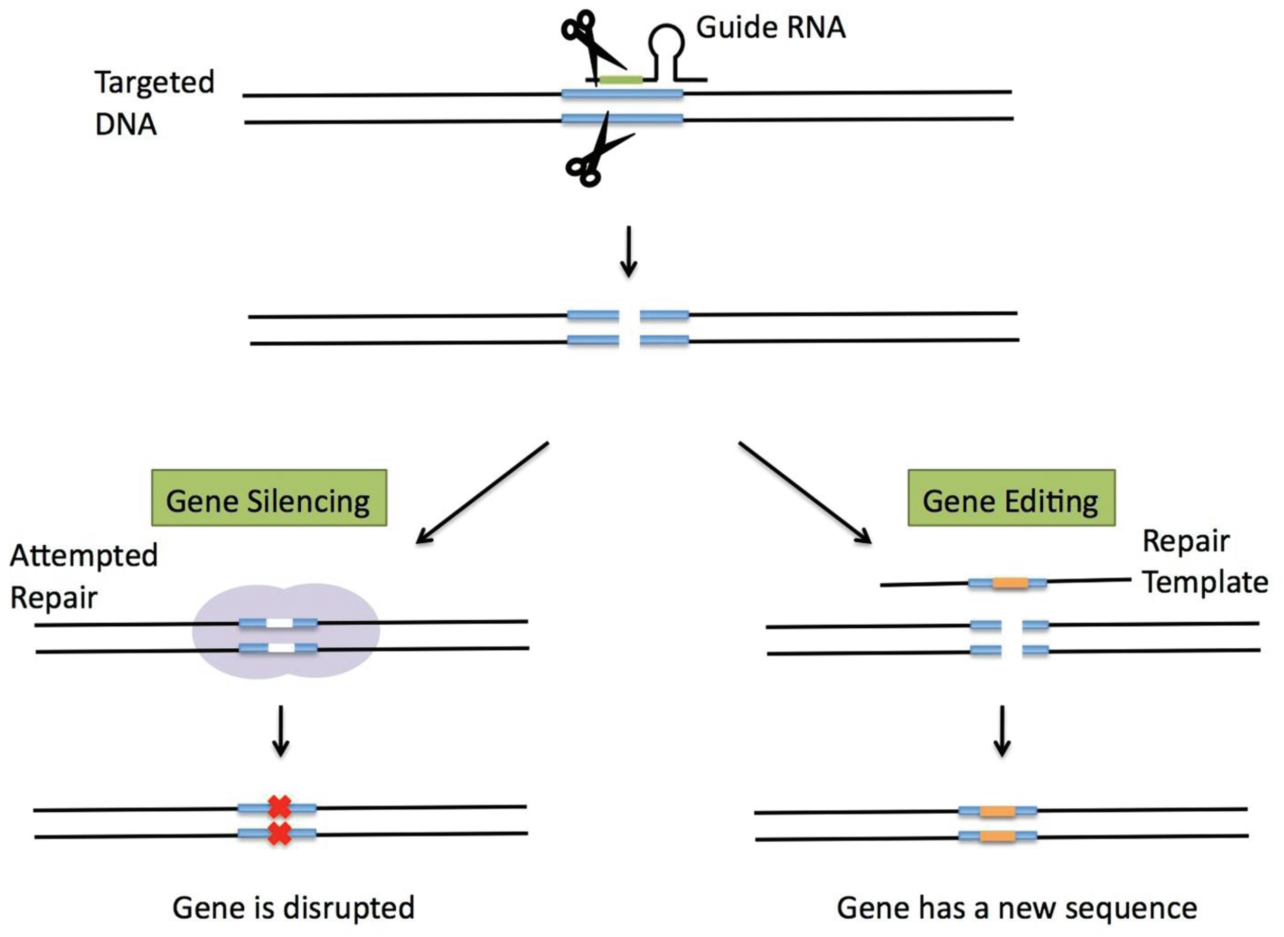
Gene silencing and editing with CRISPR. Guided RNA directs molecular machinery to cut both strands of the targeted DNA. During gene silencing, the DNA is broken and the gene is inactivated. For gene editing, a repair template with a specified sequence is added and incorporated into the DNA. The targeted DNA is now altered to carry this new sequence. (Data from Pak, 2014)

Currently used antibiotics tend to be broad spectrum, leading to in-discriminable killing of beneficial commensal bacteria and the evolution of drug resistance. CRISPR/Cas systems have been successfully used to targeted virulence factors and antibiotic resistance genes in bacteria and, as such, constitute an appealing option for the development of programmable and sequence specific antimicrobials (Bikard and Barrangou, 2017). CRISPR systems are a crucial component of the immune system of simple organisms. They are able to cut up any viral DNA sequences resulting from a viral attack. CRISPR technology is considered to be the discovery of the century in biotechnology, permitting a whole new field of gene-editing for therapeutic purposes including the development of engineered antimicrobials (Figure 1). The technology can be used to create antimicrobials whose spectrum of activity is chosen by design and to efficiently kill a target bacterial population when delivered by phage capsids both in vitro and in vivo. In addition, resistant bacteria can be re-sensitized to an antibiotic. In order to bring these strategies to the clinic, specific therapeutic approaches will have to be established. The unique advantage of CRISPR-based antimicrobials over all other strategies is their ability to kill bacteria based on their genetic sequence. This should prove advantageous in cases where it is desirable to eliminate only a select group of bacteria within a species, something that would be hard to achieve with incumbent strategies. CRISPR-based approaches will also address two grand challenges currently associated with antibiotics, namely (1) to prevent the indiscriminate eradication of intestinal bacteria that might be beneficial and (2) to lessen the selective pressure for resistance by allowing the non-target population to thrive and occupy the ecological niche. CRISPR-based technologies will open new avenues to control the composition of microbial communities rather than the traditional use of broad-spectrum antibiotics. Owing to the modularity and simplicity of CRISPR/Cas engineering, libraries of multiplexed RNA-guided nucleases can be rapidly constructed to simultaneously target antibiotic resistance and virulence determinants and to modulate the composition of complex microbial communities. This technology will reinvigorate the pipeline for new antimicrobials (Bikard and Barrangou, 2017; Kim et al., 2017).

### Antibiotic Conjugates for Enhanced Antimicrobial Efficacy

Antibiotic conjugates are increasing used as a targeted therapy for the treatment or prevention of several bacterial diseases (Cal et al., 2017). Antibiotics in their natural form have limitations associated with bioavailability, toxicity, and biodistribution, as well as efficacy. A problem with the use of antibiotic therapy is often related to the inability to target specific moieties. Due to these limitations, and since free antibiotics have fast and short-acting effects, several daily high doses are required to maintain therapeutic concentrations at specific locations. This, in turn, can result in concomitant damage of commensal microbiota. The possibility of using conjugates to avoid antibiotic resistant pathways has the potential to revitalize antibiotics that have lost effectiveness against resistant bacteria. Conjugation of antibiotics provides a novel means for the delivery of antibiotic to any specific tissue in the body.

### Bacteriophages as Antimicrobial Agents Against Major Pathogens

**Figure 2.**
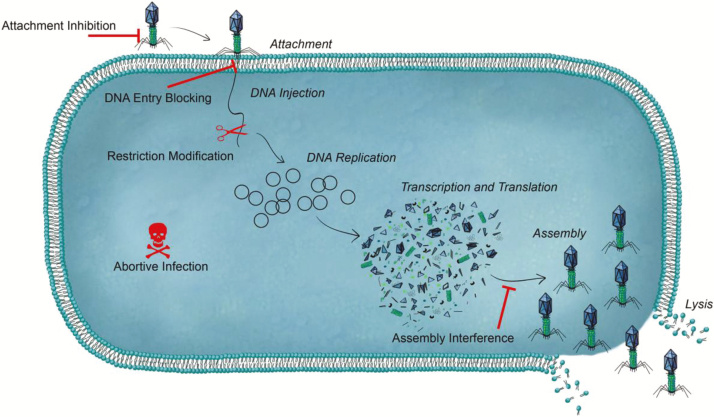
An overview of a phage attack on a bacterium and sites (red) of bacterial defense systems against phage attack. CRISPR gene-editing system can target each resistant site resulting in the ability of phage to kill bacteria. (Data from Seed (2015).)

Bacteriophages (phages for short) are viruses that can affect and kill bacteria. Bacteriophages infect bacterial cells with high specify, and in the case of lytic phages, they disrupt and lyse their host cells, resulting in cell death (Figure 2). Tail penetration through cell walls drives insertion of phage DNA into the cytoplasm of the host. Once inside the cell, specific enzymes encoded by the phage genome are synthesized to divert the host cell’s DNA and protein synthesis toward the generation of new phage particles. At a precise time at the end of the phage cycle, phage-encoded holins form pores in the cell membrane resulting in rapid cell destruction. Lytic phages have the ability to replicate exponentially and can rapidly eliminate bacteria regardless of their antibiotic resistance profiles.

Virulent phages are very appealing candidates for use as biotherapeutic agents for treatment of acute infection in animals caused by both Gram-positive and Gram-negative bacteria (Endersen et al., 2014). Orally administered phages generally reduce the intestinal pathogen concentration or cause mortality. Some of the problems previously associated with the use of phages in animals are that they have a narrow range of hosts resulting in a limitation for their use for broad spectrum protection, administered phages may induce an immune response in the animal body, bacteria may become resistant to phages, and, finally, phages are not stable at the low pH of the stomach (pH ~2) but are almost completely stable at the pH of the large intestine (around pH 6.8) (Zhang et al., 2015). Colom et al. (2015) showed that encapsulation of phages in liposomes resulted in significantly longer periods of phage retention (several days longer) in the cecum of chicken. Park et al. (2017) has recently developed a genetic engineered phage-based delivery system as an antimicrobial against *Staphylococcus aureus*. They were able to overcome the current shortcomings in phage-based delivery systems such as inefficient delivery, narrow host range, and potential transfer of virulence genes. This same system can be adapted for use with other important pathogens. Recent research developments will allow the field to continue to address concerns regarding phage therapies and finally unlock their significant potential as antimicrobial agents. Additional research must be carried out to demonstrate the health/safety concerns of engineered phages including their ability to vector antimicrobial resistance.

### Engineered Antimicrobial Peptides for the Control of Microbial Diseases In Vivo

Antimicrobial peptides are promising next generation antibiotics that hold great potential for combating bacterial resistance. Antimicrobial peptides are small amphipathic peptides (29 to 42 amino acids) that are cationic (positively charged) and have direct and indirect antimicrobial activity against Gram-positive and Gram-negative bacteria, fungi, and viruses (Li et al., 2017; Pachón-Ibáñez et al., 2017). They induce rapid killing and display a lower propensity to develop resistance than do conventional antibiotics. Despite significant progress in the past 30 yr, no peptide antibiotic has reached the clinic yet. Unfortunately, some disadvantages including stability, susceptibility to proteolysis, low activity under physiological conditions, and high cost of production must be circumvented before these peptides will reach the market place. Lam et al. (2016) synthesized a new class of antimicrobial agents, termed “structurally nanoengineered antimicrobial peptide polymers.” They exhibit sub-micromolar activity against all Gram-negative bacteria tested, while demonstrating low toxicity. Overall, structurally nanoengineered antimicrobial peptide polymers show great promise as low-cost and effective antimicrobial agents. They may be effective in combating the growing threat of resistant Gram-negative bacteria. Further research must be carried out to confirm the safety and efficacy of engineered antimicrobial peptides.

### Killing Bacterial Pathogens Using Chemically Synthesized Virus-like Nanoparticles (Nanoantibiotics)

Antimicrobial (host defense) peptides and their synthetic mimics have emerged as promising candidates for the killing of pathogenic bacteria. Cationic charge and amphiphilicity were identified as the two key antibiotic traits that help many antimicrobial peptides disrupt bacterial membranes via synergistic hydrophobic and charge interactions. Direct use of antimicrobial peptides is hindered by their expense, toxicity, and limited tissue distribution. Since the activity of antimicrobial peptides relies on their overall physicochemical property rather than their specific composition, much interest is put on developing synthetic peptides. A central dichotomy of synthetic antimicrobial peptides persists in that their hydrophobicity, which may be critical for antimicrobial activity, may also cause toxicity to mammalian cells.

The prevalent wisdom on developing membrane active antimicrobials is to seek a delicate cationic–hydrophobic balance. Jiang et al. (2017) studied the antibiotic role of nanostructures by designing spherical and rod-like polymer molecular brushes that mimic the two basic structural motifs of the bacteriophage tail. The synthetically produced tail brushes were involved in the binding of the particle to bacterial cells. They demonstrated that, while the individual polymer molecular brushes are hydrophilic and a weak antimicrobial, amphiphilicity is not a required antibiotic trait once nanostructures come into play. The nanostructured polymeric molecular brushes induced pore formation (lethality) in bacterial but not in mammalian membranes. The sizes and shapes of the nanostructures further helped to define the antibiotic activity and selectivity of the polymer molecular brushes against different families of bacteria. This study highlights the importance of nanostructures in the design of membrane activity with high activity, low toxicity to eukaryotes, and target specificity. This research has resulted in the development of an entirely new class of chemically synthesized antimicrobial that will not become antibiotic resistant and can target different types of bacteria. The new antibiotic, among other possibilities, could potentially be used as a growth promotant when added to feeds in a manner similar to the current widespread use of antibiotics. C. Conrad (<http://www.chee.uh.edu/faculty/conrad>) from the University of Houston is of the opinion that this exciting new antimicrobial could become a critical new tool needed to fight drug-resistant bacteria which may not be killed by other means. Jiang et al. (2017) stated that “they are cautiously optimistic that the rational design of nanoantibiotics with optimal activity, selectivity, biocompatibility, and biodistribution is possible by bioengineering.”

### Host Defense/Innate Immunity

The immune system is composed of an innate (non-specific) and an adaptive (specific) response. Innate immunity is constitutively present and is mobilized immediately following infection. Innate immunity is termed non-specific because the protective response is the same regardless of the initiating infection. In contrast, the adaptive immune system is slower, responds specifically, and generates an immunological memory. If the body’s first line of defense—the innate immune system—is unsuccessful in destroying the pathogens, after about 4 to 7 d the specific adaptive immune response sets in. This means that the adaptive defense takes longer, but it targets the pathogen more accurately. Enhancement of the efficacy of the immune system of animals will reduce the use of antibiotics.

### Vaccines as Important Agents to Address Antibiotic Resistance

Vaccinating humans and animals is a very effective way to prevent them from becoming infected and thereby the need for antibiotics. Making better use of existing vaccines and developing new vaccines are important ways to tackle antibiotic resistance and to reduce preventable illness and deaths. Subunit (Bobbala and Hook, 2016; Kachura et al., 2016; Garg et al., 2017), DNA (Endmann et al., 2014; Liao et al., 2017), and RNA (Reichmuth et al., 2016) vaccines are attractive alternatives to whole pathogen immunization. Garg et al. (2017) reviewed the use of a novel combination adjuvant for animal and human vaccines. Adjuvants are crucial components of vaccines as they reduce the amount and number of doses required to elicit effective immunity. Garg et al.’s (2017) three-component adjuvant contained a toll-like receptor agonist, either poly: cytosine (poly-c) or CpG oligodeoxynucleotide, a host defense peptide and polyphosphazene. Synthetic poly-c and CpG oligodeoxynucleotides are well-known potent adjuvants that enhance immune responses. The second component, host defense peptide, is a derivative of a natural peptide, which is a cationic (positively charged) amphipathic and has immunomodulatory properties. The third component, polyphosphazene, is a synthetic water-soluble biodegradable polymer with immunostimulatory properties and forms non-covalent complexes with viral and bacterial antigens to enhance their immunogenicity and stability. The trivalent adjuvant is stable and highly effective in mice, rats, pigs, sheep, chickens, and koalas and when used with various antigens, induces effective long-term humoral and cellular immunity. Katchura et al. (2016), using a different approach, used a CpG-rich oligonucleotide linked to a sugar polymer to form a soluble nanoparticle adjuvant (DV230-Ficoll). A single immunization of a recombinant anthrax antigen plus DV 230-Filcol induced high antibody titer in monkeys and completely protected them from a lethal anthrax challenge. DV230-Filcol should be an attractive adjuvant for use in vaccines, since it stimulates the rapid production of antibodies in response to a single immunization.

A radically different approach to vaccination involves the direct introduction into animal tissue of a plasmid containing a DNA sequence capable of expressing specific antigen(s) in-situ against which an immune response is obtained. Endmann et al. (2014) demonstrated that a cationic lipid formulated DNA vaccine against hepatitis B virus elicited a response in pigs of the same magnitude as a licensed protein vaccine, Engerix-B. Liao et al. (2017) produced charged (cationic) DNA polyplex vaccine microneedles. They demonstrated that the immune response was 3.5-fold greater than that seen with conventionally administered DNA. Their new microneedles vaccine has long lasting immunity, can be produced inexpensively, is easily administered to animals, has a long shelf life, and can be adapted for use in the control of almost any pathogenic disease in different animal species. More recent approaches have used mRNA to formulate vaccines. Although DNA and mRNA vaccines share many similarities, the main differences between the two vaccines is the target location for the delivery of the oligonucleotides. DNA therapeutics has to reach the nucleus, while for RNA therapeutics, the cytosol is the target. mRNA vaccines have been shown to elicit a potent immune response including the production of high concentrations of antibodies (Reichmuth et al., 2016). Lipid nanoparticles as a vector for delivery of mRNA to the cells can be easily synthesized, can protect the mRNA against degradation, and can be co-delivered with an adjuvant. RNA vaccines, like DNA vaccines, are not considered by the United States Food and Drug Administration as being gene therapies. The many advances in vaccine technology have provided a means of producing high and sustained antibody titers in animals that are relatively inexpensive and highly effective in controlling or preventing animal diseases. Additional research must be carried out with regards to the safety and efficacy of RNA and DNA vaccines before they are widely used in domestic livestock and poultry.

### Chicken Egg-Yolk Antibodies and Plant Immunoglobulins for the Control of Intestinal Pathogens in Domestic Animals and Humans

Oral immunotherapy (passive immunization) with antibodies is a highly attractive and effective approach for the control of enteric diseases due to their high specificity, effectiveness and rapidity of action. Oral administration of antibodies, derived from mammalian serum and colostrum and even monoclonal antibodies, have been used successfully but they are prohibitively expensive.

**Figure 3.**
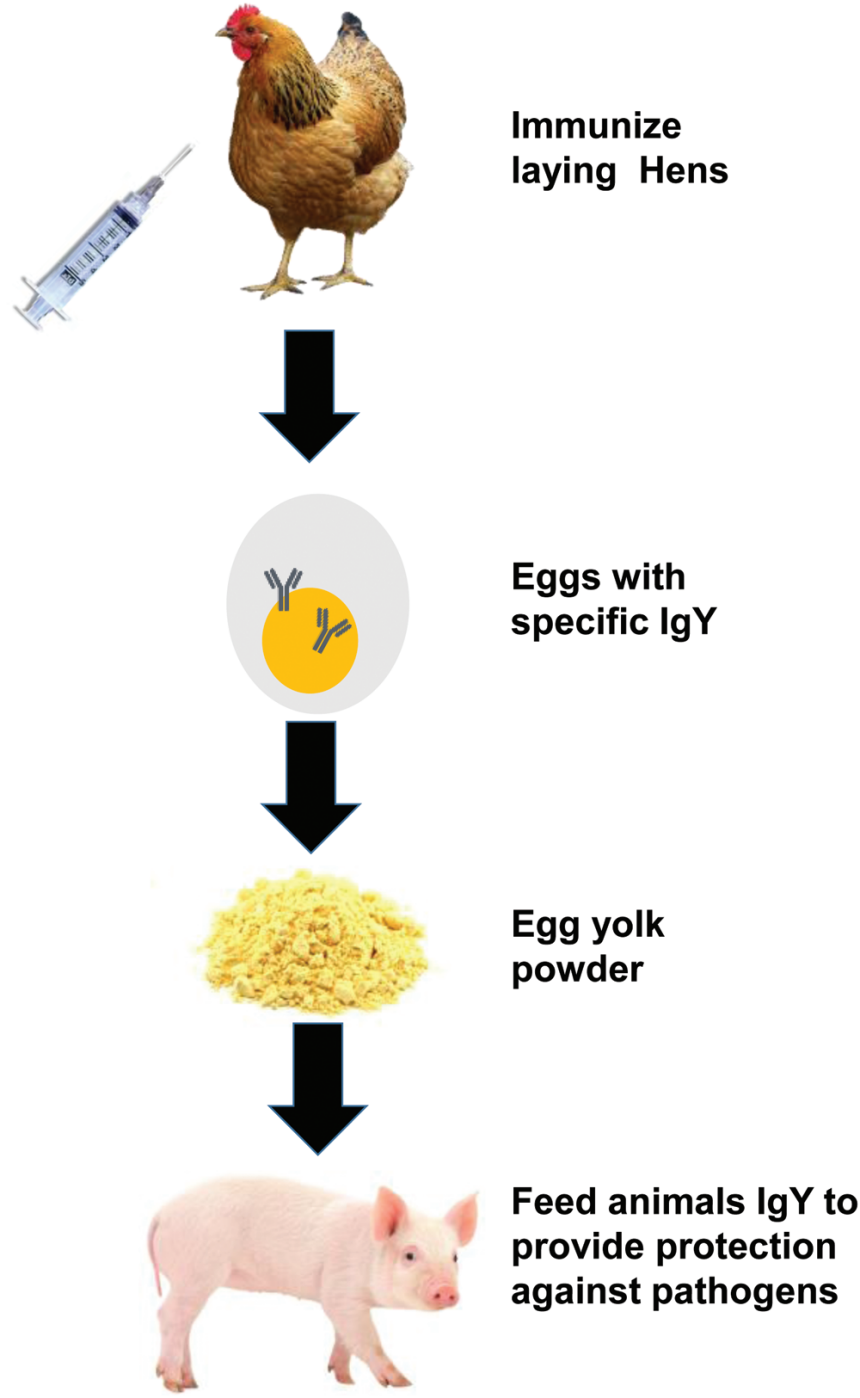
Passive immunization with hyperimmune egg-yolk antibodies as a prophylaxis and treatment of intestinal microbial diseases in domestic animals. Laying hens are immunized with a microbial virulence factor or an adhesion antigen that can be produced recombinantly in an in vitro high expression system. The immunized hens produce antibodies against the immunogen and transfer the antibodies into the egg-yolk. The antibodies in the egg or the yolk can be prepared in dry form by spray- or freeze-drying and incorporated into the diet. Egg-yolk antibodies have been shown to be highly effective at small dietary inclusion rates for the specific control or prevention of many different intestinal pathogens.

In contrast, chicken egg-yolk immunoglobulin, referred to as immunoglobulin Y, has attracted considerable attention to prevent and control disease as it possesses many advantages compared with mammalian immunoglobulin G including cost-effectiveness, convenience, and high yield of antibody (Figure 3). Orally administrated immunoglobulin Y has been used to prevent or treat a large number of bacterial and viral diseases in mammalian, avian, and aquatic species (Kovacs-Nolan and Mine, 2012). Diraviyam et al. (2014) carried out a meta-analysis on the ability of orally administered immunoglobulin Y to control diarrhea in domestic animals. The principal finding was that immunoglobulin Y was able to reduce the incidence of diarrhea across all classes of animals analyzed including pigs, mice, poultry, and calves. They suggested that more intensive animal experiments be carried out to further confirm the efficacy of immunoglobulin Y using immunoglobulin Y alone or in combination with other alternative strategies.

Although relatively small amounts of immunoglobulin Y have been shown to be highly effective in the control of intestinal pathogens (Yokoyama et al., 1992; Marquardt et al., 1999), additional research needs to be carried out to increase the efficacy of antibody treatment. This includes obtaining information on the amount of specific antibody (antibody titer) required to neutralize varying severity of infection and the required duration and frequency of the treatment. In addition to refining procedures to optimize immunoglobulin Y treatment protocols in animals, it is also important to use an immunization procedure that will maximize antibody production in laying hens as this is directly related to operational profitability and cost of the product. Several new vaccination protocols can be used to produce high concentrations of antibody in the egg-yolk of laying hens. In order to maximize the immune response obtained by immunization with the antigen, it is necessary to use an adjuvant as a component of the vaccine as it stimulates faster, stronger, and longer lasting immunity. Two novel vaccine platforms, as discussed in the section on vaccines, can be used in laying hens to produce a high and sustained production of immunoglobulin Y (Kashura et al. 2016; Garg et al., 2017). Another possibility is to use DNA (Endmann et al., 2014; Liao et al., 2017) or RNA (Reichmuth et al., 2016) vaccines as they can provide an inexpensive means of producing antibodies in eggs.

A very attractive alternative source of antibodies is to produce them in plants (Buyel et al., 2017; Edgue et al., 2017). Recent breakthroughs in biotechnology have made it possible to produce antibodies in plants on a very large scale (Buyel et al., 2017; Edgue et al., 2017). The advent of plant-based transient expression systems allows the rapid and safe production of antibodies, ranging from laboratory scale expression to industrial scale manufacturing. The key features of plant-based production include safety, speed, low cost, and convenience, allowing newcomers to rapidly master the technology and use it to its full advantage. The use of plants for product development offers the power and flexibility to easily co-express many different genes allowing the construction of novel bionanomaterials. The plant can be used to produce antibody-based supramolecular structures that will have many applications (Buyel et al., 2017). For example, it will be possible to design antibodies that will be resistant to the effects of pH and digestive enzymes in the gastrointestinal tract, thereby, reducing the amount required to be effective. Future antibody products are expected to fully capitalize on the unique features of plant-based systems. Several Global companies have the capacity to produce bionanomedicines.

### Encapsulation of Antibodies to Prevent Their Inactivation in the Gastrointestinal Tract

Oral passive immunization may be one of the most valuable applications of antibodies. However, in order to be effective, the antibodies must survive the gastrointestinal environmental and reach the target areas with their biological properties intact. As with any protein, antibodies are susceptible to proteolytic digestion. The activity of immunoglobulin Y is reduced or destroyed by peptic digestion and the low pH (around 2.0) in the stomach. In contrast, immunoglobulin Y is fairly stable against the action of proteases found in the small and large intestine where the pH is around 6 to 7. Studies on the passage of bovine immunoglobulin G through the gastrointestinal tract of human have demonstrated that only 4 to 19% of the antibody activity survived (Kelly et al., cited by Kovacs-Nolan and Mine, 2005). Alustiza et al. (2016) evaluated the in vivo protective efficacy of immunoglobulin Y in a nanocomposite matrix when used against enterotoxigenic *Escherichia coli* in challenged piglets. The protected immunoglobulin Y completely prevented enterotoxigenic *E. coli* induced diarrhea in challenged piglets, whereas the incidence of diarrhea in challenged piglets given the non-protected immunoglobulin Y was 100%. These studies demonstrated that encapsulation of immunoglobulin Y can greatly increase its effectiveness.

In summary, immunoglobulin Y is highly effective in the control of many enteric pathogens in nearly all animal, poultry and aquatic species, even though a large percentage of the antibody is inactivated in the stomach. Encapsulated immunoglobulin Y is delivered intact without degradation into the large intestine, the site at which it becomes biologically active. Research must be carried out to identify optimal amount of immunoglobulin Y required to treat a disease condition in order to increase efficacy and cost-benefits of using antibodies. Also, new vaccination strategies should be used to increase the concentration of immunoglobulin Y in the yolk of the egg. The ability to inexpensively produce antibodies in large amounts (tonnes) in plants is a promising new approach to produce antibodies for the control of intestinal diseases in animals and humans.

### Oral Treatment with Low Concentrations of Lipid Encapsulated Zinc for the Prevention and Treatment of Intestinal Diseases Caused by Pathogenic Organisms

Dietary supplementation of piglet diets with 1,500 to 3,000 ppm zinc oxide has been widely used to suppress bacterial adhesion and invasion, thereby preventing loss in weight gain, atrophy of the absorptive villi, and diarrhea. However, high concentrations of dietary zinc oxide cause environmental pollution. In Europe, the maximum level of zinc oxide that can be fed is 150 ppm, far below the concentration required to obtain a beneficial response. Recent studies by Kim et al. (2015) have demonstrated that 100 ppm of lipid encapsulated zinc oxide when added to diet was not only as effective as 2,500 ppm of native encapsulated zinc oxide in alleviating diarrhea and growth retardation in weanling pigs challenged with enterotoxigenic *E. coli*, but that it was also similar to results obtained with antibiotic treatment. These results demonstrate that lipid encapsulated zinc and probably several other modified zinc products can be effectively used at low concentrations to mitigate the effect of enterotoxigenic *E. coli* in pigs. It has also been shown that these same modified zinc compounds can be used to control other enteric diseases in most animal species including humans.

### Eubiotics as Growth Promotants

Recently, the animal feed industry has used Eubiotics as replacements for antibiotics as growth promotants. Eubiotics are mainly used to maintain intestinal eubiosis in farm animals to improve health status and performance. Dhama et al. (2014) reviewed in detail the benefits of using non-antibiotic growth promotants (Eubiotics) including the use of probiotics, prebiotics, organic acids, exogenous enzymes, essential oils, and herbs. The authors concluded that probiotics and organic acids offer the best viable alternatives to antibiotics as growth promoters. Moreover, by exploiting new genetic approaches, it should be possible to strengthen or create novel probiotics which will have unique oral immuno-therapeutic applications.

### Genetic Resistance to Diseases in Animals

Interactions between genotype and the environment of animals include genetics and health. Differences in breed susceptibility have been reported for several animal diseases including Sarcocystis infection, Foot and Mouth disease, and African Swine Fever in pigs. There is also evidence of within-breed variation for several diseases in pigs including African Swine Fever, Atrophic Rhinitis, Aujeszyky’s, Porcine Circovirus Associated disease, Salmonella and enterotoxigenic *E. coli*, F4 and F18.

Enterotoxigenic *E. coli* expressing the F4 (previously known as K88) fimbriae is a major cause of diarrhea in neonatal and pre-weaned piglets, which leads to considerable economical loss in the pig industry. Ren et al. (2012) described the causality of the MUC 13 gene for susceptibility/resistance to enterotoxigenic *E. coli* F4ac in pigs. They demonstrated that the MUC13A allele was completely associated with the F4ac non-adhesion phenotype across diverse pig populations. All susceptible animals from a broad panel of pig2 breeds carried at least one MUC13B allele. Overall their findings have important practical consequences and will have immediate impact on pig breeding programs, as they will allow the rapid elimination of the susceptible allele and consequently will greatly benefit animal welfare and the pig industry.

Similar studies with other animal diseases will greatly facility the selection of animals that are resistance to other enteric diseases. These developments will not only reduce the demand for antibiotics but will overcome the problem of antibiotic resistance with regards to specific diseases.

## Conclusions

Recent dramatic progress in biotechnology and the use of nanostructured materials have resulted in the development of new procedures to not only target antibiotic resistance bacteria but to effectively treat and prevent microbial diseases in all classes of livestock and in humans. These developments include: (1) the ability of CRIPSR/Cas gene-editing systems to target and eliminable antibiotic resistant genes in bacteria and to directly kill microorganisms; (2) production of bacteriophages that are lethal to bacteria in vivo; (3) use of engineered peptides and chemically synthesized virus-like mimics capable of efficiently killing microorganisms; (4) use of new and modified vaccination technologies as agents to address antibiotic resistance, and (5) the use of egg-yolk and plant immunoglobulins, and encapsulated zinc oxide to rapidly and efficiently treat intestinal pathogens in humans and animals. It is anticipated that most of the above procedures will result in the production and marketing of safe, new, and efficient antimicrobial products having different modes of action, most of which, should not induce microbial resistance. These advances will help address the problem of antibiotic resistance significantly.

## About the Authors

**Dr Ronald. R. Marquardt** has been awarded Professor emeritus status at the University of Manitoba. He was a professor for over 30 yr. His research was in the area of Nutritional Biochemistry, Molecular Biology, Toxicology, and Analytical Chemistry. Specific egg-yolk antibodies from laying hens, immunized with recombinant produced antigens, were shown to be highly effective in the control of food and feed-borne pathogens. Dr Marquardt has published over 300 peer-reviewed research papers. His score on Research Gate is high, which is a reflection of the high impact of his research.

**Dr Suzhen Li** came to Canada from China as a visiting scientist in the Department of Animal Science, University of Manitoba in 1991. Her research involved the development of diagnostic assays for mycotoxins using mono- and polyclonal antibodies; and the pharmacokinetics of ochratoxin A in rodents. Her research has been published in high impact international journals. She obtained her Ph.D. in 1999 from the University of Manitoba. Her Postdoctoral Research was in the field of DNA technology. She was a co-founder of All Natural Nutritional Products (ANNP) Inc. in 2005. ANNP produces and markets nutraceutical products.
